# Transactions of the Gynæcological Society of Chicago, Regular Meeting, December 16, 1887

**Published:** 1888-04

**Authors:** 


### SOCIETY REPORTS.

### TRANSACTIONS OF THE GYNÆCOLOGICAL SOCIETY OF CHICAGO.

REGULAR MEETING, DECEMBER 16, 1887.

The President, HENRY T. BYFORD, M.D., in the Chair.

The PRESIDENT exhibited

A NEW UTERINE ELEVATOR, and said: I have been in the habit, for a long time, of introducing an ordinary straight, hard-rubber intrauterine stem into the retroflex uterus before replacing it, in order to stiffen or straighten it, and to serve as an indicator of the position of the fundus after it has ascended out of reach. Last summer a cutler showed me a uterine elevator, invented by Dr. Miller, of San Francisco, which consisted of a straight steel stem, fastened upon the end of a thimble, with the end in view of making the stem a continuation of the finger end. I was unable to use this one, because in bending my finger so as to push the cervix back in place, my knuckle would catch against the posterior vaginal wall or pelvic floor. I therefore constructed the instrument which I show you. There are three stems—a jointed steel stem, like that upon the end of Emmet's elevator, and two copper ones of different sizes, slightly flexible. Any of these may be attached to a shovel-shaped piece in which the finger end lies at right angles to the stem. The cervix may thus be pushed backward or sidewise, and the fundus pried forward. With the finger thus against the end of the stem, and practically against the cervix, we can calculate the position of the fundus, the amount of resistance to replacement, and avoid all violence and danger.

The SECRETARY read the following

REPORT OF A CASE OF TUMOR OF THE ILEUM; DEATH FROM INTESTINAL HÆMORRHAGE; EXTENSIVE COMPLICATING LESIONS.

Reported by Dr. F. W. MERCER.

The following history was communicated to me by Dr. Converse, the attending physician:

Annie W., single, æt. 34 years, was always considered in good health. Had menstruated regularly without undue pain till within the past year, when it was observed that the flow was not quite as copious as in health. All the other bodily functions had been regularly performed so far as the friends knew. Miss W. was of cheerful disposition, vivacious, and physically strong, being able to toss her sister's child, weighing about twenty pounds, up at arm's length above her head.

About one year ago she consulted a leading physician of this city, to whom she complained of a throbbing sensation in the left inguinal region, and also of some slight digestive disorder. The doctor informed her that he considered the throbbing due to the presence of gas in the intestine, and prescribed some digestive. After this the patient went along as usual, not making any special complaint, till the morning of October 16, 1887, when she said she felt tired, reclining upon the bed in her room. About 11 a. m. she had a movement of the bowels, consisting almost entirely of blood, but not large in amount. Dr. C., who examined the stool, concluded it was due to "bleeding piles." Absolute rest was ordered. At 12.30 p. m. the patient left her bed and entered the bath-room, where she fainted while passing another bloody stool. She was carried back to bed, where she soon revived. Dr. C. saw her again, and ordered pyrogallic acid and ergot, with brandy at short intervals. From this time till 9 p. m. she grew very restless, having involuntary discharges of blood, which were received upon cloths, making any accurate estimate of quantity impossible. At about the last-named hour I was called in consultation, and found the patient tossing from side to side of the bed, groaning and complaining of pain in the umbilicus. The features looked shrunken and pinched, the surface blanched and clammy, the face very cold to the touch, and the pulse barely perceptible. In fact, the patient appeared moribund from hæmorrhage.

An examination of the abdomen was made, and the presence of a tumor, quite symmetrical, of globular form, was discovered in the median line, just above the pubes; an enlarged uterus was suspected as the source of the hæmorrhage, and an examination per vaginam was made, with the result that the os was found to be of pin-point character, cervix elongated, and giving no indications of hæmorrhage.

Hot-water bags and bottles were applied to the surface, warm blankets packed about the body, and brandy given subcutaneously, with aromatic spirits of ammonium by the mouth. Transfusion was thought of, but, the means not being at hand, it was not tried. Dr. E. W. Sawyer was called, but little could be done, as the patient was now *in articulo mortis.*

Section, fourteen hours after death; Dr. E. D. Converse, Dr. E. W. Sawyer, Dr. W. Barry, Dr. Frank T. Andrews, and your reporter being present. The section was made by Dr. Frank T. Andrews. The body appeared well nourished; the rigor well marked. Upon reflecting the abdominal parietes, the intestines appeared rather pale, and not unduly distended. At the lower hypogastrium a tumor was found resting in the median line. It was globular, ten centimetres in diameter, and weighed four hundred and fifty-four grammes; was moderately firm in consistence, and attached by a slender pedicle to the small intestine (ileum), about fifteen inches above the cæcum. Upon section of the ileum, an oval opening was discovered upon its mucous surface, about four millimetres by three millimetres. This opening corresponded to the attached pedicle, and communicated directly with an artery of about four millimetres calibre. It was undoubtedly from this that the fatal hæmorrhage occurred. There were old and extensive adhesions of the intestines to the peritoneum. Both the right and left kidney showed a greatly reduced cortex; and the tubuli and pelves were found filled with pus. The liver was very fatty and friable, breaking under pressure like old granular tallow. The uterus was converted into an irregular lobulated mass, consisting of fibroid tumors. The thorax was not opened.

Dr. F. W. MERCER: I very much regret that I have not the stained specimens to show you to-night. I have, however, examined the tissues, and the tumors of the uterus are intramural myomata. The tumor of the intestine is also a myoma. It is a very vascular structure. I have been unable to develop the literature on this subject; few, if any, such cases have been reported, and I have not been able to find a parallel case to the intestinal tumor, as regards the peculiarity of its attachment and the openness of the blood vessels. Myomata are often very vascular.

Dr. E. W. SAWYER: As a still further emphasis of the condition of this patient, I want to say that she filled a difficult clerical position, involving a good deal of labor, up to the day previous to her death. I have seen her in life for the last ten years, and knew her to be industrious and a healthy appearing woman.

Professor C. T. PARKES: It might be proper to suggest the idea that this was an angioma. The partial description that has been given would call my attention to growths of that character: its extreme vascularity; the size of the vessels entering into it; its position, differing in this respect from myoma. The tissue about the walls might be connective tissue, showing some evidence of muscular fibre.

I speak merely from my experience. I have seen quite a number of myomata, but never saw any that showed the peculiarities of this specimen. It might have developed, as suggested by Dr. Dudley, in some other place, this being a foreign position. As to its attachment, I think it would be in reason to say that it might show muscular fibre if it was an outgrowth from the intestine. I would not say, of course, that it is not a myoma.

Professor A. REEVES JACKSON: It seems remarkable that this should be a myomatous tumor when so little muscular structure can be found as a basis in the intestine. The case, too, is interesting and remarkable in its history, from which we learn that the woman had degeneration of long standing of a portion of the liver; that there were adhesions of the intestines to the abdominal wall, indicating the former existence of peritonitis; that there was a fibroma of the uterus; and yet, despite all these diseases, the patient was in good health up to a recent date. All this is very remarkable. I know that women can live and seem fairly healthy with a good deal of disease present, but such an amount as was present in this instance seems quite inconsistent with a condition of *good* health. It is exceedingly important that this specimen should be examined microscopically, because its nature must be of very great interest to the pathologist.

On motion, the subject was referred for further investigation of the tumor to a committee, consisting of Drs. Fenger, Mercer, and Sawyer, and their report is to be read at the January meeting.

Professor E. C. DUDLEY reported

A CASE OF VAGINAL HYSTERECTOMY FOR SARCOMA UTERI.

The patient, a multipara, 62 years of age, came to me from Dr. Sibree, of Sturgeon Bay, Wis. Dr. Sibree, several months before, had removed a soft, friable mass filling the uterus and vagina. When the case came to my clinic at St. Luke's Hospital two months ago, the tumor, which had returned, enormously distended the uterus and vagina. Under ether, the operation previously performed by Dr. Sibree was repeated, and a soft, friable mass, weighing not less than two pounds, was removed. The tumor was attached by a short pedicle, about three-fourths of an inch in diameter, to the left side of the uterus, about on a level with the os internum. The specimen was examined by Dr. Wing, who pronounced it to be a sarcoma; I therefore determined to remove the uterus per vaginam, which was done three weeks ago to-day.

The case is remarkable and interesting. Remarkable, because sarcoma of the uterus is a somewhat rare disease, the number of cases on record being less than one hundred. Interesting, because a method of operation was employed which has heretofore not been very much used—the method of Péan, *i. e.*, hemostasis was secured entirely by means of pressure-forceps, no sutures or ligatures having been used. The cervical canal was first stuffed with absorbent cotton, and closed with a single suture, which was passed through the anterior and posterior lips. This was done in order to prevent any of the contents of the uterus from coming in contact with the perineum in case it was found necessary to turn the cervix into the pelvic cavity. The cervix, seized with strong lock vulsellum forceps, was drawn to the vulva, and an incision was made with scissors entirely around the cervix at the utero-vaginal attachment; with the finger the post-cervical structures were torn away from the cervix, keeping close to the uterus until the cul-de-sac of Douglas was reached; with two fingers in the cul-de-sac of Douglas, it was easy to enlarge this opening by tearing, until I had reached the region of the broad ligament on either side; I then attempted to divide anteriorly in the same way, but, being solicitous about invading the bladder, I kept so close to the uterus as actually to tear up a layer of the muscular structures. I had carried this process beyond the vesico-uterine attachments, indeed almost to the fundus, before I discovered that I must be beyond the region of the bladder. I did not at once enter the anterior cul-de-sac, but, taking a pressure-forceps with long blade in the right hand, I passed the index finger of the left through the posterior opening, and hooked the point of the finger around the left broad ligament; then, with the lower blade of the forceps in the posterior opening, I punched through to the finger tip with the other blade, and locked the forceps, thus securing the left broad ligament. The right broad ligament was secured in the same way, and the utero-vesical attachment severed with the scissors and the uterus removed. Upon putting the forceps upon the right broad ligament, and finding that the disease had extended through the uterine wall on that side and involved the uterine end of the right broad ligament, I pulled the ligament out and put on another pair of forceps back of those originally used. These forceps included the ovary and Fallopian tube on that side, and I hope all the disease.

The only difficulty in this operation was in consequence of extensive adhesions of the uterus posteriorly to the small intestine. At least twenty minutes were consumed in carefully breaking up these adhesions. The right ovary and tube came down and were secured in the grasp of the forceps, but, the patient having passed the menopause, the left were not removed. Several bleeding points anterior and posterior to the uterus were secured by means of ordinary pressure-forceps, all of which were left in the vagina.

Instead of closing the abdominal wound with sutures, the peritoneal edges, anteriorly and posteriorly, were caught at two points with lock-forceps, so that in reality the peritoneal wound was closed at these points, leaving space enough between the forceps for drainage. All the forceps, except those on the broad ligaments, were removed in twenty-four hours; these were removed at the end of forty-eight hours. It would possibly be safe to remove even the forceps from the broad ligaments in twenty-four hours, but leaving them forty-eight hours is an additional safeguard, and insures more prompt separation of the necrosed tissues within their grasp.

The patient had no bad symptom until the fourth day after the operation, when she developed a slightly elevated temperature and a pulse of 140, very weak and almost like the pulse of collapse; but with a moderate amount of stimulation, circulation greatly improved, and was again normal in twenty-four hours. It is now three weeks since the operation; she has had no further trouble and is sitting up. I learned after this trouble with the circulation what I had not recognized before and what would have deterred me from operating had I known it, that the patient had a fatty heart.

I think this method of removing the uterus will be generally adopted. The operation in this case lasted forty minutes. If it had not been for the adhesions it could have been done in twenty minutes without difficulty.

This operation may have a wider field than vaginal hysterectomy; I have determined that the next case I have of uterine myoma, in which supra-vaginal hysterectomy would ordinarily be performed, to open the abdomen, lift the tumor out through the abdominal wound, and then, instead of using the serre-nœud, to secure the broad ligaments by means of lock-forceps in the vagina. It would probably be easy, by having the index and middle fingers in the pelvic-cavity, one on either side of the broad ligament as a guide, to force the blades of the forceps through close to the uterus on either side of the ligament to the finger tips, and then, having secured both ligaments, sever the anterior and posterior uterine attachments. The peritoneal edges of the vaginal wound might then be closed with a continuous catgut suture, or seized with lock-forceps in the vagina, as already described. This method of performing hysterectomy for myoma when the tumor is too large to be delivered through the vagina is worth trying; it would enable the operator to dispense with all extra-peritoneal methods of hemostasis, and might afford all the advantages which belong to intra-peritoneal hemostasis for ovariotomy.

The PRESIDENT reported

A CASE OF VAGINAL HYSTERECTOMY, and exhibited specimen.

Miss P. is a virgin, 57 years of age; ceased menstruating over ten years ago. About eight months ago she had a slight hæmorrhage from the uterus, and for five months before the operation had been bleeding (sometimes profusely) most of the time. She had been bedridden for five weeks, suffered frequently with severe pain in the lower part of the abdomen, and was the most waxy, anæmic looking person I remember to have seen. Her pulse ranged from 100 to 120. Her evening temperature, from the normal in the morning, ran up to 101° F., and sometimes higher upon the few evenings before the operation. When not bleeding she had an offensive vaginal discharge. She was subject to daily hysterical attacks, in which she and her friends feared she might die. A loud anæmic cardiac murmur could be heard. Vaginal indagation revealed a friable, vascular mass about the size of a small hen's egg, projecting from the cervix into the vagina, and continuous with the posterior lip. A piece taken from the lower end was examined and pronounced papilloma. I had considered it cancer and had contemplated hysterectomy, but felt relieved at the diagnosis, because the operation would have been long and difficult on account of the virgin and senile condition of the vagina and perineum, and the success would have been doubtful by reason of the extreme anæmia and nervous prostration. I also feared she would not bear anæsthesia well for the necessary length of time.

She was anæsthetized at 9 a. m., December 7, for the purpose of curetting, but the posterior wall of the cervix as far as the internal os was found so degenerated that a complete removal was considered impossible without breaking into the cul-de-sac of Douglas. The posterior vaginal wall was also infiltrated behind the cervix. I therefore resolved to take out the uterus, and did so at 1.30 p. m. The method I employed proved so successful and seemed so well adapted to her case that I think it worth while to mention it and advise it for cases presenting similar difficulties.

After a thorough disinfection of the parts, I introduced suture of juniper catgut around the vaginal fornices so as to make a circle of ligatures. I made a bloodless incision inside of the circle, commencing in front and separating the bladder and the broad ligaments for a short distance at the sides before introducing the posterior sutures, completing the circular incision posteriorly, and opening the peritoneal cavity. Scarcely half an ounce of blood was lost. After opening the cul-de-sac its entire width, a pair of hemostatic forceps was placed on the left sacro-uterine and base of the left broad ligament, and the uterus cut loose on that side as far as the forceps reached. The manœuvre was then repeated on the other side. Then another pair of forceps was placed on each side just above the first, and the tissues severed as high as they extended. A third pair on each side included the Fallopian tubes, and enabled me to cut out the uterus with the loss of less than an ounce of blood except that which had oozed from the diseased cervix. Some iodoform gauze was stuffed in between the forceps. The forceps were removed at the end of twenty-eight hours, with great distress to the patient and followed by a temporary rise of temperature from $99\frac{2}{5}°$ to $100\frac{2}{5}°$ F. The temperature went down, however, and did not rise that high again until the beginning of the fifth day, when it again went up to $100\frac{2}{5}°$ F., but subsided upon removal of the tampon, which had become offensive. She turned on her side at the beginning of the fourth day. The bowels were moved on the fifth day by a Seidlitz powder. After the removal of the iodoform gauze on the beginning of the fifth day, vaginal douches, with a tube for the return flow, were used twice a day. After the sixth day they were carbolized.

Having performed the operation both ways, viz., with and without hemostatic forceps, I feel justified in asserting that ligatures are better for cases in which the size of the vagina and mobility of the uterus allow of their application without too much loss of time, for there is less bruising of the broad ligaments and less sloughing afterward. They may be left long and used instead of sutures to draw the stumps together over the vaginal opening. For cases in which time is an important element, and the broad ligaments can not be rapidly ligated, the forceps are preferable. In the hands of the beginner they are safer, because they may be applied with less handling and exposure of the peritoneum, and are not liable to be followed by hæmorrhage. Ligation of the vaginal walls, before cutting, can be done so quickly and easily that it is a desirable procedure in all cases.

The growth, as you see, has extended as high as the internal os; with the main part of it taken off, it looks like an epithelioma of the cervix. The uterus is small in size. Sarcoma, originating in the cervix, would seem to be a rare occurrence, and this one case is, as far as I can determine, the thirteenth one recorded. One has been recorded by G. Veit (*Handbuch der Speciellen Pathologie*, etc., von Virchow); one by Scanzoni (*Lehrbuch der weiblichen Sexualorgane*); one by Kunert (*Archiv für Gynekologie*, Bd. VI, p. 113); one by Leopold (*Archiv f. Gyn.*, Bd. VI, p. 493); one by Grenser (*Archiv f. Gyn.*, Bd. VI, p. 501); two by Spiegelberg (*Archiv f. Gyn.*, Bd. XIV, p. 178, and Bd. XV, p. 437); one by Rein (*Archiv f. Gyn.*, Bd. XV, p. 187); one by Winckler (*Archiv f. Gyn.*, Bd. XXI, p. 309); one by Schwartz (*Beerman, Inaug. Diss.*, Göttingen, 1876); one by Zweifel (*Centralblat für Gyn.*, 1884); Hunter (*Am. Journal of Obstetrics*, Vol. XVII, p. 523), and this one. Some of these were not primary sarcomas, but were developed secondary to other growths, such as papilloma, fibroma, etc.

REPORT OF PATHOLOGIST.

*Microscopical Examination of the Cervix.*—On the surface the growth presents the structure of papilloma, *i. e.*, villous projections consisting of a connective-tissue stroma covered over completely by several layers of ovoid cells. Upon the surface of these there is a single layer of columnar epithelium.

In the specimen this has been stripped off on part of the surface.

The columnar epithelium is not as tall and less delicate than that of the normal mucosa.

*Normal Mucosa.*—At the base of these villi the tissues are considerably altered. They consist largely of granulation cells. The normal glandular elements have quite disappeared in some parts; in others they are greatly modified, the cells lining the follicles being irregular, ovoid, or spindle-shaped.

Many of the glands are quite replaced by sarcoma corpuscles.

In some points of the specimen there occurs a delicate network of branching cells, presenting the characteristic appearance of myxoma. These portions are considerably softer than the surrounding structure.

*Diagnosis.*—Papilloma at surface.

Myxo-sarcoma at base.

The disease was probably primarily simple papilloma, and is now in the stage of transition to sarcoma.

MARIE J. MERGLER.

Professor J. H. ETHERIDGE: I understood Dr. Byford to say that a portion of the growth involved the vagina also, and I would like to ask him if he could follow up the broad ligament well enough to find if there was any involvement of that structure.

Dr. BYFORD: I did follow it up and put on the forceps.

Professor ETHERIDGE: I saw a case about two weeks ago that was very interesting and instructive to me. It was the removal of the uterus for cancer of the cervix. Upon examination, at first we thought we would not take it out, but as the whole cervix was involved and it had spread over upon the sides of the vagina, the left side a very little and considerably upon the right side, it was concluded to go on and do the operation. The operator freed the cervix from the vagina and then commenced the peeling up process; the broad ligaments were exposed, and the forceps put on, and the uterus cut away. In the left broad ligament, throughout its whole extent to the pelvic wall, there were nodular enlargements, showing that the broad ligament itself was involved, and that, too, upon the side in which there was the least encroachment of the vagina; on the right side there was no involvement of the broad ligament. There were eight or nine forceps left in the vagina, which were removed at the end of forty-eight hours without any trouble. She has recovered from the operation nicely.

I have often wondered how the broad ligament appeared when infiltrated with cancerous material, and I think now that I know.

If the case Dr. Dudley reported is the one I saw, and he did not speak of one thing, I would like to speak of it, and that was a clever bit of ingenuity on the part of a clever operator in retroflexing the uterus. If I remember rightly, he attempted to push it down and draw it down from beneath without avail, and then, by taking a pair of vulsellum forceps, he grasped the uterus just above the vaginal attachment and drew it down, then took another forceps and drew it down a little more, then another, and so on, and in that way got the uterus down easily; which impresses one who has had trouble in getting the uterus down within reach.

Since the October meeting, at which a paper of mine on this subject was read by title, another case has passed through my hands which I have not reported to this society. It was a case in which there was a development of a small fibroid tumor in the posterior uterine wall. All attempts at retroflexing or retroverting the uterus were unavailing, and it had to be removed by detaching the broad ligaments which the uterus turned upward. The patient has gone along without trouble and has made a complete recovery. The uterus was removed for incoercible hæmorrhage. Everything else had been tried—scraping, stimulating applications, ergot, etc.—and the operation which was expected to be performed was the removal of the ovaries. But as I had had the experience of removing two pairs of ovaries for bleeding fibroids, and they kept on bleeding, I explained to her the difference between the removal of the uterus and the ovaries, and she finally accepted the removal of the uterus.

I believe one of the greatest advantages of the forceps over the ligature is the superior facility for free drainage. All fluids will run down through the opening in the vagina left by the forceps in a perfect manner, and there is no danger of the fluids remaining in the peritoneal cavity by the closing of the top of the vagina, which is speedily brought about. After the patient is put to bed it is but a few hours before we have a closed cavity in which fluids may decompose and septic peritonitis ensue, unless effective drainage, such as the forceps afford, be used. I think that one advantage over the ligature is enough to induce every operator to use the forceps.

Professor JACKSON: It occurred to me, in examining this specimen, that it was not necessary to remove the entire uterus. High amputation of the cervix would have removed all the disease and would have lessened the danger to the patient.

I want to refer to a point in Dr. Dudley's remarks with reference to the attachment of the sarcoma to the uterus by a pedicle. I desire to ask whether that is a usual method of attachment of a sarcoma? In the cases of uterine sarcoma that I have seen the disease commenced on a flat surface—the attachment was sessile. I once saw a case which I at first supposed was one of sarcoma, but subsequent examination with the microscope determined that it was not a sarcoma but a degenerated fibrous polypus.

These microscopic examinations of malignant disease, by the way, are not always reliable. In my own experience I have received three widely different reports, all made by competent persons and from the same specimen, so that I must confess I have less confidence in the microscope as a diagnostic instrument than I formerly had. I regret this very much because, in many cases, we depend almost entirely on such investigations to determine our diagnosis and treatment.

Dr. PARKES: I was exceedingly interested in Dr. Dudley's case, and especially in the manner of securing the broad ligaments by means of forceps. I had the pleasure of witnessing the use of these forceps by the person who first invented them, Péan, of Paris. In 1883 I did the first operation that has come under my care for removal of the uterus per vaginam by this method. In 1886 I did it again, and during the early part of this year I removed three uteri, and in each of them I employed the method that has been described this evening, the use of the snap-forceps in controlling the broad ligaments, instead of ligatures. After listening to a report of similar cases by Dr. Etheridge, I remarked that I did not see why, in this method, the uterus should be retroverted; that so far as my experience went I found no difficulty, after the division was made in the cul-de-sac of Douglas, in reaching the top of the broad ligaments with my finger, and found no difficulty in severing the attachment, and had no subsequent difficulty from hæmorrhage. I can very well see that occasional cases will be met with where it will be difficult, if not impossible, to reach the top of the broad ligament so as to be sure that every portion is included in the grasp of the forceps, as in Dr. Byford's case, and in another case I saw where the uterus had in its posterior wall a large tumor, and where it was with great difficulty removed. In these instances it may be necessary to reverse the uterus. The objection to reversal is that it twists the broad ligaments, and we know that any instrument used for pressure acts better the thinner the tissue that is engaged in the forceps.

It is only lately that very much has been said in the journals about the use of forceps instead of ligatures. Within the last two years an article has appeared in the journals by a French gentleman who has reported some forty cases operated upon in this way. The objection that comes to my mind in the use of these forceps in operation for myomata of the uterus and large size, is that in the cases I have seen the broad ligament has been carried up with the uterus to such height above the top of the vaginal wall that no forceps I have seen would embrace all the tissues. Still the plan would be good; abdominal section would enable one to tie the broad ligaments half way down to the uterine arteries, and then the forceps could be used.

Professor E. C. DUDLEY, in closing the discussion, said: Dr. Jackson has asked for my experience relative to the question whether sarcoma is apt to be attached to the uterine wall by a pedicle. Sarcoma of the uterus is too rare to permit any one operator to speak from experience. It is, however, true that sarcoma may be attached to the uterine wall by a broad or narrow pedicle ; on the other hand, as Dr. Jackson says, it may be intramural. This tumor had a very short pedicle ; the growth filled and distended the entire uterus; there was perhaps a pound of sarcomatous tumor in the uterus at the time of my first operation, three or four weeks before the second.

I have more confidence in the accuracy of pathological observations through the microscope than Dr. Jackson, never having had occasion to question the diagnosis of a pathologist, although, like Dr. Jackson, I have frequently employed two or three microscopists to examine the same specimen independently, but I have always taken care to employ good pathologists, of whom Dr. Wing is one.

The objection of Dr. Parkes against the operation which I have indicated for removing myomata and securing the broad ligaments by means of lock-forceps in the vagina would not render the procedure impracticable. The broad ligaments could easily be stripped from the uterus until it became possible to include them in the grasp of the forceps. For this operation, it would be well to have, in addition to the ordinary forceps, four pairs of forceps with very long blades, two of which could be fastened on either broad ligament close to the uterus, and the ligaments could be divided between them; then the ordinary forceps might be passed through the vagina with their blades on either side of the remaining portions of the broad ligaments. After removing the uterus, the entire broad ligament on either side could then be grasped in a single pair of vaginal forceps, which should have longer and stronger blades than for ordinary vaginal hysterectomy on account of the great hypertrophy of the ligaments.

Dr. Byford considers the method of Péan inferior to that of the ligature for certain cases in vaginal hysterectomy. Péan's method seems to me better and safer for all cases. The hemostasis can be more quickly secured by the forceps than by the ligature. A single pair of forceps will secure a mass which would require several ligatures. The hemostasis is almost absolute when secured by the forceps, but is uncertain with the ligature. After the forceps have been left on for forty-eight hours, there is necrosis of that portion of the ligaments within their grasp and we thereby get rid of it; not so with the ligature mass, it must remain as a slough, sometimes for many days. Moreover, after a ligament is found to be involved in the disease for which the uterus is being removed, it may be easily drawn out by means of the forceps first applied, the surrounding tissues stripped off, and another forceps applied back of the first ones. This would be very difficult, sometimes impossible, with the ligature. The mortality of this operation may perhaps be reduced as low or lower than that of ordinary ovariotomy. It is certainly an easier operation than ovariotomy with adhesions. The hemostatic forceps have indeed changed a very formidable operation to a very simple one.

Retroverting or inverting the uterus through the opening posterior or anterior to the uterus and bringing the fundus into the vagina is objectionable. First, some of the contents of the uterus may get into the peritoneal cavity and make trouble. Second, the body of the uterus in the vagina fills it so full as to leave very little space in which to complete the operation. It will always be well, however, as a precaution against infection of the abdomen from the malignant disease, to tampon the uterine canal with cotton and close the os externum with a suture, so that if it become necessary to retrovert or antevert the uterus nothing can get out. Iodoform gauze was used as a vaginal tampon in this case; when removed in twenty-four hours, it was found to be fœtid, and therefore seemed rather an element of danger than of safety. It also interfered with drainage. If used at all, it should be placed between the forceps and the vesico-vaginal septum, where it would not be so liable to obstruct drainage as if placed between the forceps and the recto-vaginal wall. It would always seem desirable to close the peritoneal edges of the wound with lock-forceps, as was done in this case. They did not close the wound enough to prevent drainage, and they do protect the patient against the danger of protrusion of abdominal viscera in case of severe retching, vomiting, or coughing.

Dr. H. T. Byford: I cannot agree with the last speaker that iodoform gauze interferes with drainage and favors decomposition. As the vagina cannot well be douched during the first few days, the secretions must accumulate until forced or drawn up through the vaginal entrance, which, in the dorsal decubitus, is the highest end of the vaginal canal. By placing a small roll of iodoform gauze well up against the stumps (drawn down by the ligatures), and then stuffing in a long narrow strip so as to fill the vaginal canal loosely and extend out to a dry piece of gauze between the labiæ, we can drain off the fluid portion of the exudations by capillary drainage, promote hemostasis, prevent prolapse of the intestines, support the base of the bladder, and keep the stumps stationary until an exudate has fixed them and closed the peritoneal cavity.

As to the advantage of the forceps in enabling us to remove more of the broad ligaments, I think that the less of the broad ligament removed the better. If the disease has extended into the ligament, the operation should not be performed. I think that the proposed combination of the abdominal and vaginal methods by the application of the hemostatic forceps from the vagina is more complicated, more difficult, and more dangerous than supra-vaginal amputation or abdominal hysterectomy. Hemostasis can be secured without it. In case the cervix must, on account of disease, be removed with the uterus through the abdomen, then the forceps might be applied from below, but the mortality would, in the nature of the case, be greater than that of vaginal hysterectomy or supra-vaginal amputation.

In answer to the criticism that a high amputation of the cervix would have been preferable, I will say that the cervical wall was so deeply invaded that the cul-da-sac would have necessarily been opened. Had it not been opened there was not enough vaginal wall to cover the raw surfaces, and the hæmorrhage that would have occurred, and the suppuration afterward, whether the cautery had been applied or not, would have been much more liable to kill this feeble patient than hysterectomy. The septicemic symptoms disappeared in a day, and the anæmia is daily diminishing. In some cases vaginal hysterectomy is the safer operation.

